# Optimal Relay Network for Aerial Remote Inspections

**DOI:** 10.3390/s22041391

**Published:** 2022-02-11

**Authors:** Luis Ramos Pinto, Luis Almeida

**Affiliations:** 1 Independent Researcher, 1350-248 Lisboa, Portugal; eng.pintoluis@gmail.com; 2 CISTER, Faculdade de Engenharia da Universidade do Porto, 4200-465 Porto, Portugal

**Keywords:** multi-hop network, packet delivery ratio, relay network, TDMA, throughput, UAV, wireless networks

## Abstract

Unmanned aerial vehicles (UAVs), in particular multirotors, are becoming the de facto tool for aerial sensing and remote inspection. In large industrial facilities, a UAV can transmit an online video stream to inspect difficult-to-access structures, such as chimneys, deposits, and towers. However, the communication range is limited, constraining the UAV operation range. This limitation can be overcome with relaying UAVs placed between the source UAV and the control station, creating a line of communication links. In this work, we assume the use of a digital data packet network technology, namely WiFi, and tackle the problem of defining the exact placement for the relaying UAVs that creates an end-to-end channel with maximal delivery of data packets. We consider asymmetric communication links and we show an increase as large as 15% in end-to-end packet delivery ratio when compared to an equidistant placement. We also discuss the deployment of such a network and propose a fully distributed method that converges to the global optimal relay positions taking, on average, 1.4 times the time taken by a centralized method.

## 1. Introduction

The versatility of unmanned aerial vehicles (UAV or *drones*) has allowed their use for multiple purposes, either recreational, engineering, scientific, or military. Many of these applications require remote vision, either for their own control with first-person-view (FPV), for aerial sensing of areas of interest, or for remote inspection of large structures that are difficult to access. This last case can be found in widespread industrial plants to inspect chimneys, silos, reactors, buildings, etc. [1]. In these situations, a UAV typically captures a video stream that is transmitted to an operator in a base station that fine-tunes the navigation and positioning of the UAV to carry out the inspection with detail. Multirotor UAVs are particularly suited to such situations because of their high maneuverability, including hovering capacity, and ad hoc communication that dispenses a predeployed network infrastructure [2]. However, the limited power and types of antenna available in UAVs pose a limit to the communication range, thus limiting the device range of operation. This is particularly relevant when using data networks, such as IEEE 802.11 (WiFi), which may improve the quality of the video transmission, and integrate other data communications, too, but exhibit relatively short range (few hundreds of meters in line-of-sight) and abrupt degradation with distance [3].

**Figure 1:**
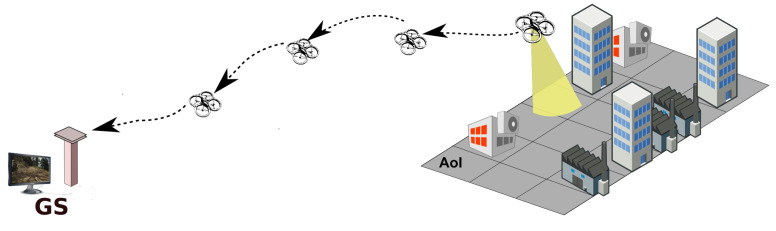
Single-source aerial stream to a ground sink using UAV relays to transmit data.

One possible way to mitigate the range limitation is adding UAV relays that can hover between the source UAV and the ground station, forwarding packets from one side to the other, forming a multi-hop line network (Figure 1). Using relays to maintain network connectivity is a well-known approach [4]. However, the placement of the relay nodes is critical since their position determines the length of the links, which, in turn, has an impact on the links quality, thus on the quality of the end-to-end network, too. In our case, the main purpose of the network is to convey video information, which is particularly affected by the loss of packets [5]. Hence, we define our problem as finding the positions of the relay UAVs that maximize the end-to-end packet delivery ratio (PDR) in the line network from source UAV to base station. To the best of our knowledge, maximizing end-to-end PDR has not been used before to drive the placement of relays in an aerial line network.

At the basis of our approach is a model of the PDR of each link as a function of the link length, which we proposed in [3] and which exhibited good adherence to experimental data. This model also assumes a few features of the medium access control (MAC) layer, namely the use of an overlay time division multiple access (TDMA) transmission control scheme to prevent transmission collisions [6], the disabling of automatic retries, and a fixed bit rate. In the current work, we consider the same assumptions, which also enable a direct conversion between end-to-end throughput and end-to-end PDR.

In the following section we discuss the related work in a broader context. Here, we refer to the previous work of the authors as motivation for the current work. A first step in addressing the problem under consideration was reported in [3] where the end-to-end network throughput was established as a function of the link’s length. The authors showed how to place the relays optimally to cover the largest distance with the minimum number of relays, while simultaneously maximizing the end-to-end throughput. Minimizing the number of UAVs is particularly relevant because it allows reducing end-to-end latency, which is crucial in the use cases we are considering, i.e., remote control of the source UAV for visual inspections. As shown in [7], keeping an end-to-end latency below 1–2 s constrains the number of relays to just a few, hardly above 5 or 6, thus scalability is not a concern. The work in [3], however, was limited to networks with homogeneous links, i.e., with similar PDR model. In such conditions, the optimal relay placement is equidistant along the source to base station line.

Naturally, the assumption of links with similar PDR model falls short in practice, since the links often exhibit asymmetric properties due to obstacles, differences in the antennas, and local interference, among other effects. These link asymmetries cause different error rates and throughput, negatively impacting the network end-to-end performance. This was shown in [7], where the authors proposed a method to compensate those asymmetries acting on the throughput, adjusting the duration of the time slots to give less time to links that have high throughput, and vice versa. This provides a fast compensation method for a network with asymmetric but fixed link properties, balancing the throughput among all network links.

However, a better asymptotic result can be achieved by adjusting the position of the relays dynamically to improve the end-to-end PDR instead, thus improving the global network channel. The work in [8] shows a preliminary incursion in this direction, analyzing the case of one relay, determining the optimal relay placement between a fixed source and base station, and validating this result in practice. On the other hand, the paper left open the formal solution to the placement problem and it addressed just the simpler case of a single relay.

In the current work, we present a general optimal solution for the referred problem of placing relays in a line network between a source node (the source UAV) and a sink (the base station) considering asymmetrical links, in particular, each link with its own PDR model. Our solution places the relays in a way that maximizes the end-to-end PDR, solving an open problem in the related state-of-the-art. Specifically, our contributions are the following:

- An optimal solution for relay placement in a line network considering asymmetric links that maximizes the end-to-end PDR. This work subsumes the work in [3] that considered symmetric links, but builds on the empirical link PDR model developed therein. Moreover, it also subsumes the work in [8] that addressed the preliminary single relay case.
- Two deployment strategies with centralized and distributed positions control that can operate dynamically, generating optimal relay positions recurrently. We provide a comparison between both in terms of convergence time and overhead, together with an empirical proof of convergence of the distributed solution to the global optimal relay positions.

The next section discusses related work available in the literature. Section 3 introduces the problem of relaying video over a line of hovering relays and shows the formal grounds for the asymmetric relay placement. Section 4 presents the optimal relay placement solution, with examples and performance assessment. Section 5 presents two deployment methods for the solution proposed before, namely a centralized and a distributed relay positions control methods. Emphasis is placed on the distributed method, its relative performance, and convergence. The online estimation of the links’ PDR model is also introduced. Finally, Section 6 concludes the paper.

## 2. Related Work

The scientific literature available related to UAVs is extremely rich, including networks of multi-UAV systems [2]. Among these, several works address the general UAVs placement problem. The placement of UAVs can be carried out for service-driven purposes, i.e., to improve the specific service provided by the multi-UAV system. For example, the placement of sensor UAVs can improve sensing area coverage [9,10], relay UAVs can improve connectivity of sensors in a wireless sensor network or in the Internet-of-Things [11,12,13], or access point UAVs can improve the coverage of users in accessing a communications infrastructure in an energy-efficient way [14,15]. Note that in these works, one or more UAVs provide service in parallel to other users/nodes.

Alternatively, the placement of the UAVs can be carried out for network-driven purposes, i.e., to improve inter-UAV communication in a multi-hop network that conveys data from users to consumers, sometimes called *backbone*. This is the general framework of our problem that differs from the service-driven approach in the purpose, placing relay UAVs to improve networking features, independently of the application. The work in [4] addresses this problem providing an optimal, but static, i.e., offline, relay placement approach, and not focusing necessarily on UAVs. They optimize network connectivity, combining inter-node reachability and network throughput.

From the point of view of the network, using relay UAVs has several advantages over ground relays since their elevated position provides better line-of-sight conditions [12]. In addition, UAVs can easily move to better locations, thus being suitable for dynamic network adaptation by means of dynamic node positioning [16].

The work in [17], similarly to ours, considers a freely moving source UAV that needs to keep a live communication connection with a base station on the ground, beyond the single link range. This connection is achieved with relay UAVs that provide multi-hop communication with dynamic relay positioning. The authors take a routing perspective and propose controlling the movement of the relay nodes so that they always provide a path between the dynamic source node and the base station. Curiously, the work uses a loss model, based on received power, which is the same for the whole network, leading to symmetrical link losses. Thus, the authors arrive at the conclusion that the best path is when all relays are aligned between the source and the base station and the relays are equidistantly placed along that line, which matches our result presented in [3] and is subsumed by our current work. The authors call their protocol elastic relay network construction (ERNetC) and acknowledge that few works in the literature address the multi-hop live connection requirement. They compare against optimized link state routing (OLSR) [18], which was developed to provide routing support to mobile ad hoc networks, and predictive-OLSR (P-OLSR) [19], which is an enhanced version for more dynamic networks of UAVs. Both show significantly worse performance than ERNetC.

Finally, the works in [20,21] address the ad hoc communication between mobile ground robots. Though not UAVs, they tackle a problem with similarity to ours that is worth referring. They also aim at setting up a line of relay nodes to convey a multimedia stream from a source robot in one extreme to a ground station on the other. However, they take advantage of specific propagation conditions inside tunnels that do not apply to the communication between UAVs. Moreover, none of them study the relays placement that provides the best end-to-end PDR assuming asymmetric link quality.

Therefore, we believe that our end-to-end PDR maximization technique based on placing the relay UAVs to compensate link asymmetries in what concerns their own local PDR model is novel and represents a contribution to the state-of-the-art in multi-hop line networks of relay UAVs.

## 3. Problem

**Figure 2:**
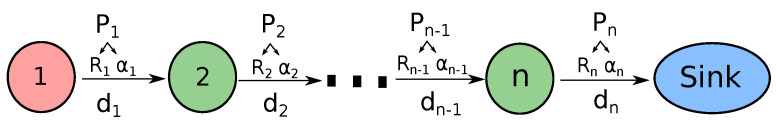
Multi-hop line network model. The PDR of link *i*, between nodes *i* and i+1, is $P_{i}$ with parameters $R_{i},\alpha_{i}$, and its length is $d_{i}$.

As referred to previously, in this work, we consider an aerial network of *n* UAVs with hovering capacity, such as multirotors, identified uniquely by their index i∈[1,n], and a ground (or control) station with index i=n+1. The UAVs form a line network topology and communicate essentially in one direction, only, from the farthest (1st) UAV, which we call the source, that generates a live sensing (video) stream, to the ground station, which we will call the sink. Although we consider unidirectional source to sink communication only, our model does not preclude low-bandwidth communication in the opposite direction, such as controls sent by the ground station to the UAVs. Note that the corresponding bandwidth is significantly lower than that of the online sensing stream, reducing the relevance of PDR optimization in that direction since reliable retransmission-based mechanisms can be emplaced. Thus, for our work, we ignore all other than the source to sink transmissions. We assume that all intermediate UAVs (i∈[2,n]) are relays that communicate with their immediate neighbors, only. This implies the existence of *n* links, i.e., node *i* receives the sensing stream from node i−1 through link i−1 and retransmits it to node *i* through link *i*. Finally, we consider the links to be physically aligned with a total network length of *L*. The constraint of physical alignment is currently relevant, since we consider that, when moving one relay, one of its links shrinks while the other one grows the same length, given the fixed total length. The case of links that can form different angles between them is left for future work. Figure 2 shows an example with *n* UAVs and a sink (in blue), in which the first UAV is the source (in pink) and UAVs 2 to *n* are relays. There are *n* links with lengths $d_{1}$ to $d_{n}$, with a total network length of $L=d_{1}+d_{2}+\cdots +d_{n}$.

In what concerns the packet delivery ratio (PDR) of each link, we use the model proposed in [3] in which a link *i* exhibits a PDR $P_{i}$ that varies with the link length $d_{i}$ according to a negative exponential law, as presented in Equation (1). Here, $\beta_{i}$ is given by Equation (2), $R_{i}$ is the link length at which the PDR is reduced to 50%, and $\alpha_{i}$ is related to the slope of the PDR curve at length R. Along the formal developments that follow, we will frequently refer to link *i* as its corresponding PDR model duplet $\left(R_{i},\alpha_{i}\right)$.

(1) $$P_{i}\left(d_{i}\right)=e^{\beta_{i}\;{d_{i}}^{\alpha_{i}}}$$

(2) $$\beta_{i}=\frac{-\log(2)}{{R_{i}}^{\alpha_{i}}}$$

Once the network model and link PDR model are defined, we can now formalize the problem at hand. In this work, our objective is to maximize the performance of the line network in what concerns the end-to-end PDR by adjusting the placement of the relay nodes along the line, or equivalently, adjusting the lengths of the network links subject to the total fixed network length *L*. Before we express this objective formally, it is important to state a few more assumptions. First, we assume that packets lost in the forwarding process are not recovered. If relevant, recovery must be managed at a higher layer. This is common with streams of real-time data, as is the case here. Moreover, we consider that the processes that generate packet losses are independent across links. This also means that the performance of each link *i* is solely dependent on its length $d_{i}$ and the medium characteristics ($R_{i}$, $\alpha_{i}$), all links being independent of each other. Finally, we assume there is a global TDMA coordination scheme in place, so that nodes transmit in their disjoint time slots, only, preventing interference among nodes transmissions.

Under these assumptions, the end-to-end PDR can be obtained by a simple multiplication of the PDRs of the individual links, as expressed in Equation (3). Here, we use d to represent the vector of *n* link lengths and $P_{\mathrm{net}}\left(d\right)$ as the end-to-end network PDR that results from applying the link lengths vector d.

(3) $$\begin{array}{cc} P_{\mathrm{net}}\left(d\right) & =\prod_{i=1}^{n}P_{i}\left(d_{i}\right)\\ d & =[d_{1},d_{2},d_{3},\ldots ,d_{n}] \end{array}$$

We can now state our optimization problem as in Equation (4), i.e., finding the vector of link lengths d that maximizes the end-to-end PDR $P_{\mathrm{net}}\left(d\right)$, subject to the fixed network length constraint *L*.

(4) $$\begin{array}{cc} P_{\mathrm{opt}}=\max_{d}P_{\mathrm{net}}\left(d\right) & =\max_{d}\prod_{i=1}^{n}P_{i}\left(d_{i}\right)=e^{\beta_{1}{d_{1}}^{\alpha_{1}}}\cdot \ldots \cdot e^{\beta_{n}{d_{n}}^{\alpha_{n}}}\\ \; & \mathrm{st}.\;L-\sum_{k=1}^{n}d_{k}=0 \end{array}$$

## 4. Global Solution

To solve the optimization problem expressed in Equation (4), we resort to the Lagrangian method for maximization. Thus, we start by expressing the corresponding Lagrangian expression A(d) as in Equation (5) and its gradient as in Equation (6).

(5) $$A\left(d,\lambda \right)=\prod_{i=1}^{n}P_{i}\left(d_{i}\right)+\lambda \left(L-\sum_{i=1}^{n}d_{i}\right)$$

(6) $$\nabla A\left(d,\lambda \right)=\left(\frac{\partial A}{\partial d_{1}},\ldots ,\frac{\partial A}{\partial \lambda }\right)$$

To solve the gradient, we start by computing the partial derivatives of the Lagrangian expression with respect to $d_{i}$, as in Equation (7).

(7) $$\frac{\partial }{\partial d_{i}}A(d,\lambda )=\left(\prod_{j=1,j\neq i}^{n}P_{j}\left(d_{j}\right)\right)\cdot \frac{d}{dd_{i}}P_{i}\left(d_{i}\right)-\lambda$$

Note that deriving $P_{i}\left(d_{i}\right)$ is straightforward since it is an exponential function, resulting in Equation (8). Thus, we can now rewrite Equation (7) as in Equation (9).

(8) $$\begin{array}{cc} \frac{d}{dd_{i}}P_{i}= & \frac{d}{dd_{i}}e^{\beta_{i}{d_{i}}^{\alpha_{i}}}=\\ \; & =\left(\frac{d}{dd_{i}}\beta_{i}{d_{i}}^{\alpha_{i}}\right)e^{\beta_{i}{d_{i}}^{\alpha_{i}}}=\left(\beta_{i}\alpha_{i}{d_{i}}^{\alpha_{i}-1}\right)P_{i} \end{array}$$

(9) $$\begin{array}{c} \frac{\partial }{\partial d_{i}}A(d,\lambda )=\left(\prod_{j=1,j\neq i}^{n}P_{j}\left(d_{j}\right)\right)\cdot \left(\beta_{i}\alpha_{i}{d_{i}}^{\alpha_{i}-1}\right)P_{i}-\lambda \end{array}$$

We can now establish the gradient of A(d,λ) as in Equation (10).

(10) $$\begin{array}{cc} \nabla A(d,\lambda ) & =\\ \; & \left(\forall_{i\in [1,n]}\left[\left(\beta_{i}\alpha_{i}{d_{i}}^{\alpha_{i}-1}\right)\prod_{k=1}^{n}P_{k}\left(d_{k}\right)-\lambda \right],L-\sum_{k=1}^{n}d_{k}\right) \end{array}$$

To solve the maximization problem, we equal the gradient to zero and solve for $d_{i}$, thus obtaining the following system of equations (Equation (11)):

(11) $$\begin{array}{cc} \; & \nabla A(d,\lambda )=0\Leftrightarrow \\ \Leftrightarrow \; & \left\{\begin{array}{c} \beta_{1}\alpha_{1}{d_{1}}^{\alpha_{1}-1}\prod_{k=1}^{n}P_{k}\left(d_{k}\right)-\lambda =0\\ \beta_{2}\alpha_{2}{d_{2}}^{\alpha_{2}-1}\prod_{k=1}^{n}P_{k}\left(d_{k}\right)-\lambda =0\\ \ldots \\ \beta_{n}\alpha_{n}{d_{n}}^{\alpha_{n}-1}\prod_{k=1}^{n}P_{k}\left(d_{k}\right)-\lambda =0\\ L-\sum_{k=1}^{n}d_{k}=0 \end{array}\right. \end{array}$$

This system of equations also means that, for any two nodes *g* and *h*, we have the following relationship (Equation (12)):

(12) βgαgdg(αg−1)=βhαhdh(αh−1)⇔⇔xxxxxxdg(αg−1)=βhαhβgαgdh(αh−1)⇔⇔xxxxxxdg=βhαhβgαg1αg−1dhαh−1αg−1

Considering that most of the terms in Equation (12) correspond to constants, we can thus rewrite the optimal links length relationship as in Equation (13).

(13) $$\begin{array}{cc} \; & d_{g}=\Psi_{g,h}\cdot \;{d_{h}}^{\theta_{g,h}}\\ \; & \;\mathrm{where}:\;\\ \; & \Psi_{g,h}\equiv {\left(\frac{\beta_{h}\alpha_{h}}{\beta_{g}\alpha_{g}}\right)}^{\left(\frac{1}{\alpha_{g}-1}\right)}={\left(\frac{{R_{g}}^{\alpha_{g}}\alpha_{h}}{{R_{h}}^{\alpha_{h}}\alpha_{g}}\right)}^{\left(\frac{1}{\alpha_{g}-1}\right)}\\ \; & \theta_{g,h}\equiv \left(\frac{\alpha_{h}-1}{\alpha_{g}-1}\right) \end{array}$$

In practical terms, to solve the system in Equation (11) we can start by computing $d_{n}$ first and then use the ratio in Equation (13) to express all other $d_{i}$ with i∈[1,n−1] as a function of $d_{n}$. This is expressed in Equation (14).

(14) $$\begin{array}{cc} \; & \left\{\begin{array}{c} d_{1}=\Psi_{1,n}\cdot {d_{n}}^{\theta_{1,n}}\\ d_{2}=\Psi_{2,n}\cdot {d_{n}}^{\theta_{2,n}}\\ \ldots \\ d_{n-1}=\Psi_{n-1,n}\cdot {d_{n}}^{\theta_{n-1,n}}\\ L=d_{1}+d_{2}+\ldots +d_{n} \end{array}\right. \end{array}$$

To compute $d_{n}$ we can take the last equation in the system and rewrite it as a function of $d_{n}$, only (Equation (15)).

(15) $$L=\sum_{i=1}^{n-1}\left(\Psi_{i,n}\cdot d_{n}^{\theta_{i,n}}\right)+d_{n}$$

Nevertheless, in the general case, Equation (15) has no closed-form solution. It is a Laurent polynomial, i.e., a polynomial with fractional (and/or negative) exponents, and the most common way of solving such polynomials is resorting to numerical methods such as a binary search. That is the approach followed in this work.

Finally, note that if the link PDR models are equal, then constants $\Psi_{g,h}=\theta_{g,h}=1,\forall_{g.h}$, which implies, from Equation (11), that the optimal network PDR (end-to-end) is achieved when all links have the same length, i.e., the relays are placed equidistantly between source and sink. This matches the results in [3], which are subsumed in our current work.

### 4.1. Examples

To illustrate the problem and solution method just described, we herein present two examples, one with two links, thus source, sink, and one relay in between, and another one with three links, thus with two relays in between. We will consider that the link models are known for all links, an assumption that will be dropped later on. The parameters of the link model were obtained randomly within ranges that were observed in practice, to preserve material consistency.

#### 4.1.1. A Case with Two Links

Consider a set of two links (n=2) defined by models $\left\{\left(R_{i},\alpha_{i}\right),i\in \left[1,2\right]\right\}$ with vectors R=[100,120] and α=[2.6,2.6], and total network length L=140m. We thus aim at finding the set of link lengths $d=[d_{1},d_{2}]$ that maximizes the end-to-end PDR $P_{\mathrm{net}}\left(d\right)=\prod_{i=1}^{2}\;P_{i}\left(d_{i}\right)$, keeping *L* constant, as stated in Equation (4).

We start by computing the constant vectors Ψ and θ as in Equation (13):

$$\begin{array}{cc} \Psi_{1,2} & ={\left(\frac{100^{2.6}}{120^{2.6}}\right)}^{1/1.6}=0.7436\\ \theta_{1,2} & =1 \end{array}$$

Then, we compute $d_{n}$ (in this case $d_{2}$) as a solution for the Laurent polynomial presented in Equation (15).

$$\begin{array}{cc} \Psi_{1,2} & \;{d_{2}}^{\theta_{1,2}}+d_{2}=\Psi_{1,2}\;d_{2}+d_{2}=L\Leftrightarrow \\ d_{2} & =L/(1+\Psi_{1,2})\approx 80.3 \end{array}$$

Knowing the optimal length of second link ($d_{2}$), we can, in this simple case, directly extract the optimal length of the first link ($d_{1}$) from the network length constraint *L*.

$$d_{1}=L-d_{2}\approx 59.7$$

The maximal end-to-end PDR $P_{\mathrm{net}}$ that can be achieved with the referred links occurs when the link lengths are $d_{\mathrm{opt}}=\left[59.7,80.3\right]$ m and its value is the following:

$$P_{\mathrm{opt}}=P_{\mathrm{net}}\left(d_{\mathrm{opt}}\right)=P_{1}\left(59.7\right)\cdot P_{2}\left(80.3\right)=0.6536$$

**Figure 3:**
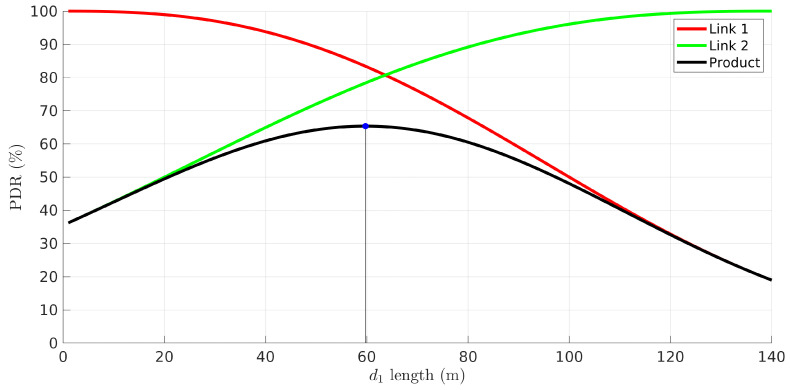
PDR of link 1 (red), link 2 (green), and their product, i.e., network (black), as a function of the length of the first link $d_{1}$ and L=140.

Generally, with *n* links we can represent the PDR solution in an *n*-dimensional graph. Thus, in this case, we can illustrate it with a 2D plot (Figure 3). The figure shows the PDR of the network (indicated as “product”) and of both links as a function of the position of the relay with respect to the source node, i.e., the length of the first link. The network length, $L=d_{1}+d_{2}$, is the position of the source, considered fixed. The network PDR has a maximum at $d_{1}=59.7$ m, contradicting, for example, the intuition that it would be maximized at the point that equalizes the links’ PDR (∼63 m).

#### 4.1.2. A Case with Three Links (Two Relays)

To show a case that already bears more complexity and goes beyond the simple solution provided in [8], we present here a situation with three links, thus using two relays. The problem is as follows. Consider a set of three links (n=3) defined by models $\left\{\left(R_{i},\alpha_{i}\right),i\in \left[1,3\right]\right\}$ with vectors R=[110,120,130] and α=[2.1,3.2,4.2], and total network length L=180 m. We now aim at finding the set of link lengths $d=[d_{1},d_{2},d_{3}]$ that maximizes the end-to-end PDR $P\left(L\right)=\prod_{i=1}^{3}\;P_{i}\left(d_{i}\right)$, keeping *L* constant (Equation (4)).

**Figure 4:**
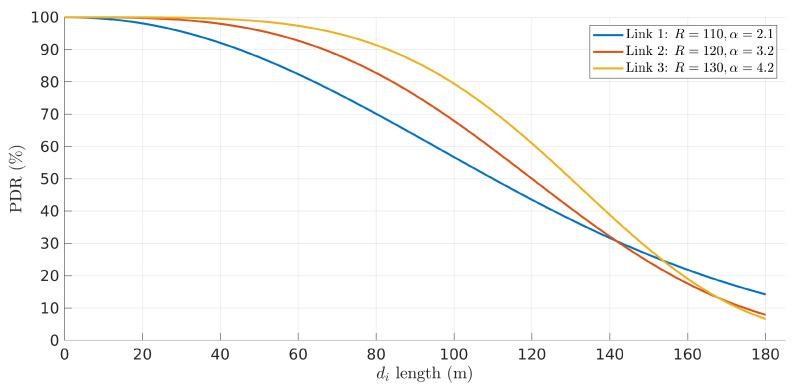
PDR of link 1, link 2, and link 3 as a function of their length.

Figure 4 represents the PDR curves of the three links. Note that, in this case, we cannot represent symmetrically the pairs of PDR curves of the two links used by each relay, as in Figure 3, since we lack one fixed node in either case; relay 1 lacks a fixed node on the right side link and relay 2 lacks a fixed node on the left side link.

Again, we start by computing the constant vectors Ψ and θ:

Ψ1,3=0.000126xxxΨ2,3=0.1102θ1,3=0.110214xxxθ2,3=1.4545

Then, we compute the solution of the corresponding Laurent polynomial (Equation (15)):

$$\begin{array}{c} \left(\Psi_{1,3}\cdot d_{3}^{\theta_{1,3}}\right)+\left(\Psi_{2,3}\cdot d_{3}^{\theta_{2,3}}\right)+d_{3}=L \end{array}$$

Applying the known constants, we obtain the following equation for $d_{3}$:

$$\left(0.000126\cdot d_{3}^{2.9091}\right)+\left(0.110214\cdot d_{3}^{1.4545}\right)=180$$

Solving this equation numerically with binary search, the value of $d_{3}\approx 77.86$ is defined. Using this value, the constant vectors Ψ and θ, and, lastly, the ratios of Equation (11), the remaining link lengths can be defined:

$$\begin{array}{cc} d_{2} & =0.000126\cdot 77.86^{1.4545}=62.17\\ d_{1} & =180-77.86-62.17=39.97 \end{array}$$

In this case, the maximum $P_{\mathrm{net}}$ that can be achieved with the referred links occurs when the link lengths are $d_{\mathrm{opt}}=\left[39.97,62.17,77.86\right]m$. The corresponding $P_{\mathrm{net}}$ value is:

$$P_{\mathrm{opt}}=P\left(d_{\mathrm{opt}}\right)=P_{1}\left(39.97\right)\cdot P_{2}\left(62.17\right)\cdot P_{3}\left(77.86\right)=0.7806$$

**Figure 5:**
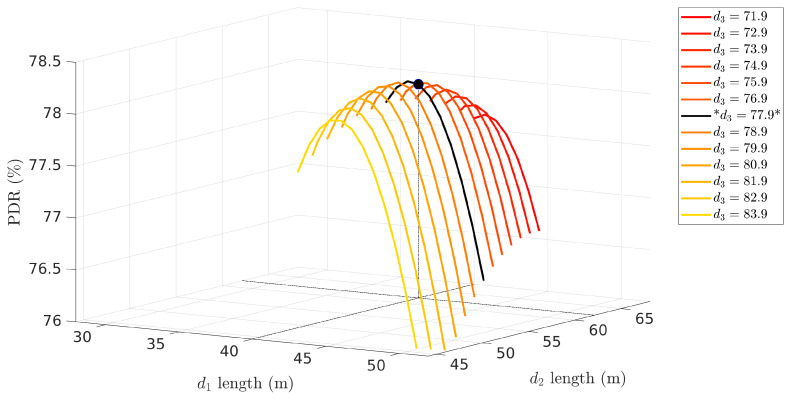
$P_{\mathrm{net}}$ (Network PDR) as a function of the length of the three links ($d_{1}$ and $d_{2}$ are represented in the horizontal plane and $d_{3}$ is a parameter shown in the legend). The maximum PDR is shown as a black dot, belonging to the black curve corresponding to the value of $d_{3}$ marked with asterisks in the legend.

With three links, we can still visualize the result with a 3D plot. Figure 5 shows the network PDR as a function of the lengths of the first two links, constrained by $d_{1}+d_{2}=L-d_{3}$. For each value of $d_{3}$ we obtain one two-dimensional PDR curve similarly to the product curve in Figure 3. The black curve represents the one that contains the highest peak, which is the maximum network PDR, achieved when $d_{3}=77.86$ m.

### 4.2. General Performance Assessment

There are benefits in optimizing the network PDR. In this section, we compare the optimal solution and an intuitive solution consisting of placing the relays at equidistant intervals between the source and the sink (the baseline). To prove such claim, we present, first, an example of a complex network with six UAVs (five relays) to observe the improvement *optimal* versus *baseline* as a function of the total network length *L*. Second, we use a vast set of randomly generated link models to achieve a general empirical characterization of the performance improvement for different network lengths and number of links.

#### 4.2.1. Performance Improvement with Six Links

**Figure 6:**
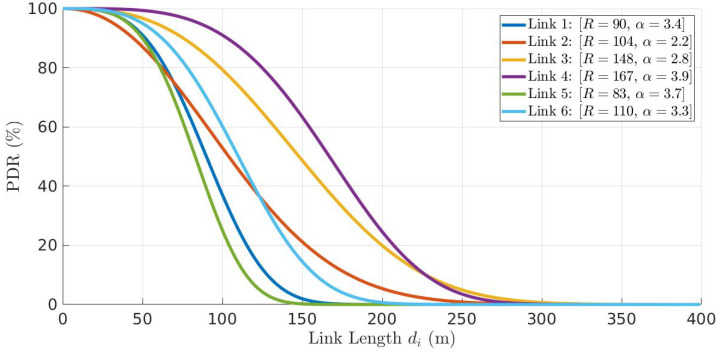
PDR models of six links as a function of link length $d_{i}$, where link *i* connects nodes *i* and i+1, ∀i∈[1,6].

Consider a network with links with different PDR models, as shown in Figure 6.

**Figure 7:**
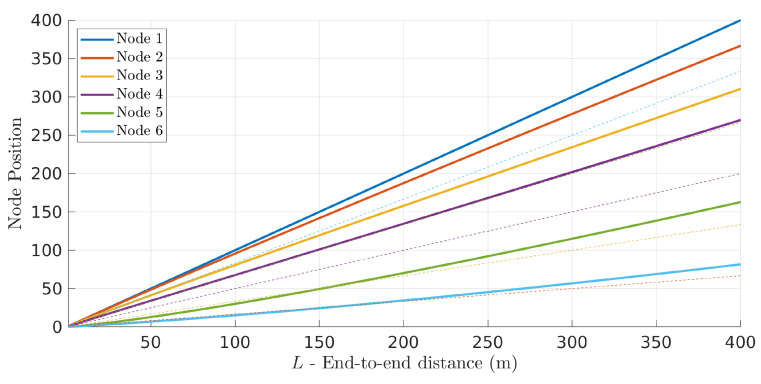
The optimal relay positions (solid lines) are different than the corresponding equidistant positions (dashed lines). Their absolute differences increase (nonlinearly) with network length *L*.

Figure 7 shows the optimal positions of the six UAVs for different network length *L* (solid lines) together with the corresponding equidistant positions (dashed lines). Node 1 corresponds to the source UAV and nodes 2 to 6 are the following five relay UAVs. Curiously, the curves also show what the path of all relay nodes would be in case they kept their optimal/equidistant positions tightly while the source (node 1) moved linearly away from the sink.

**Figure 8:**
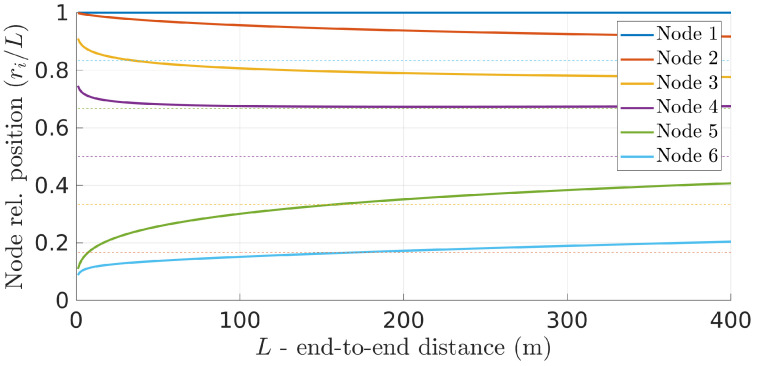
The relative positions of the relays as fractions of the total network length change with the network length itself.

The absolute difference between optimal and equidistant positions increases nonlinearly with network length. This can be better observed when plotting the relative positions of the relays concerning the fraction of the total network length instead of the absolute positions (Figure 8). The figure clearly shows that the optimal relative positions are not kept constant as the network length increases. This means that applying simple rules to maintain the relative position of each relay to their neighbors is not optimal as the network changes its overall length. In this case, the optimal positions have to be dynamically recomputed.

**Figure 9:**
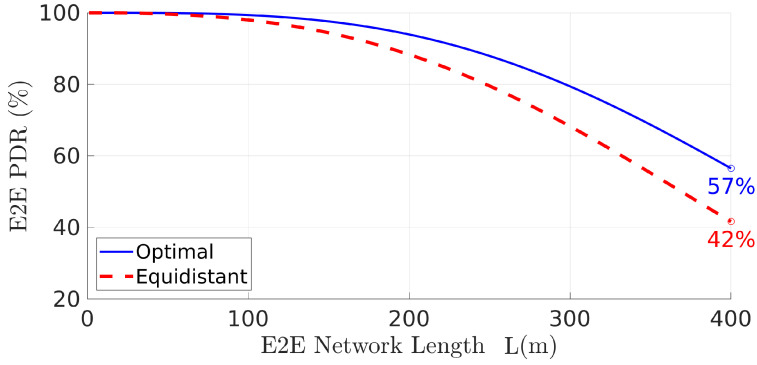
PDR $P_{\mathrm{net}}$ improves significantly when the optimal placement is used instead of equidistant positions, especially for larger network lengths.

Looking now into the network PDR achieved with the optimal relays placement we can see a significant improvement when compared to what would be achieved with an equidistant placement instead, especially when the network increases its length. This is shown in Figure 9. In this particular example, when the network is 400 m long, using the optimal solution increased the PDR by 15 percentage points, regarding equidistant placement. This represents a performance improvement of 35% (from P=42% to P=57%). Note that the network (end-to-end) PDR when the relays are placed equidistantly, i.e., $P_{\mathrm{equi}}=P_{\mathrm{net}}\left(d_{\mathrm{equi}}\right)$ with $d_{\mathrm{equi}}=\left\{d_{i}=L/n\;,\forall i\in \left[1,n\right]\right\}$, can be computed using Equation (16).

(16) $$P_{\mathrm{equi}}=P_{\mathrm{net}}\left(d_{\mathrm{equi}}\right)=\prod_{i=1}^{n}P_{i}\left(L/n\right)$$

**Figure 10:**
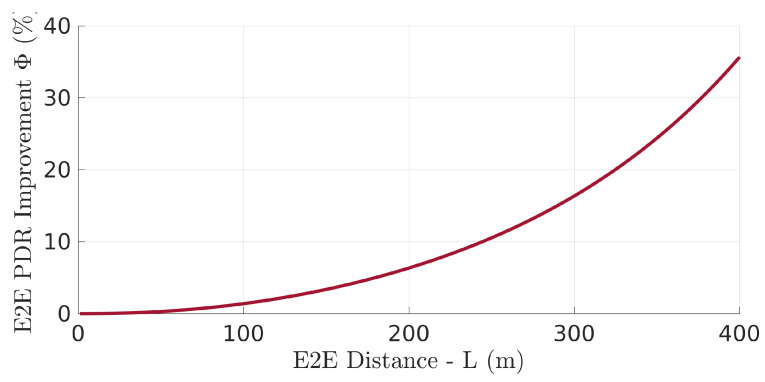
PDR $P_{\mathrm{net}}$ relative gain (Φ) using the optimal placement with respect to equidistant positioning.

The performance improvement, in network PDR, as a function of the network length *L* is shown in Figure 10. In particular, we show the relative PDR gain Φ (Equation (17)) represents the additional PDR obtained with the optimal relay positioning relative to the network PDR obtained with equidistant relays.

(17) $$\Phi =\frac{P_{\mathrm{opt}}}{P_{\mathrm{equi}}}-1=\frac{P_{\mathrm{net}}\left(d_{\mathrm{opt}}\right)}{P_{\mathrm{net}}\left(d_{\mathrm{equi}}\right)}-1$$

Figure 10 highlights the growing PDR improvement with growing network length. As we will see further on, this behavior is observed with any number of relays in the network. Increasing the number of relays significantly decreases the average length of the links, which also increases the end-to-end network PDR. However, more relays increase network delay. In general, we would be interested in using the least number of relays possible, which implies operating the network in the PDR region in which the PDR starts to drop. This is also the region where the optimal relay placement starts to make a difference, highlighting the relevance of this work.

#### 4.2.2. General Characterization with Random Links

To find a general characterization of the PDR improvement that can be achieved with the optimal relay placement, we generated 10,000 random networks with random link models, varying number of relays and network length, and observed the PDR gain Φ as defined in Equation (17).

**Figure 11:**
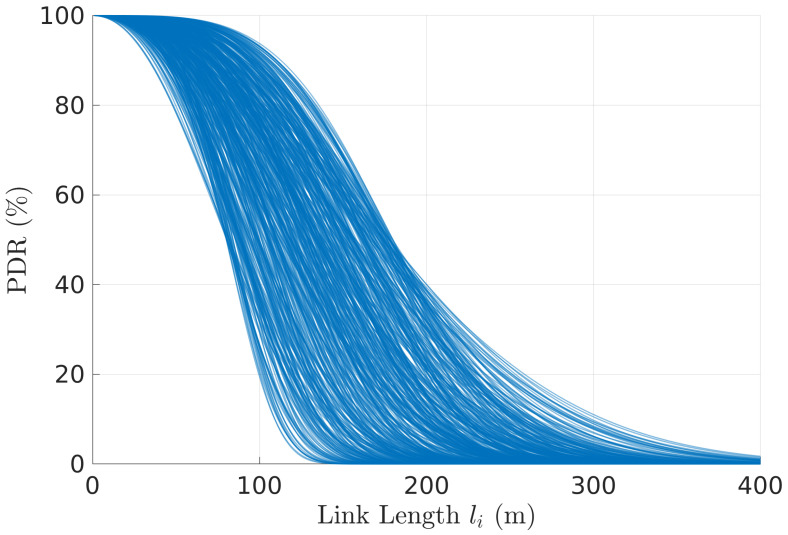
Pool of link PDR models (R,α) used for the general performance characterization (R∈[80,180] and α∈[2.1,4.2]).

We started by generating a vast pool of links, where each link is generated with a random pair (R,α), taken uniformly from R∈[80,180] and α∈[2.1,4.2], ranges that we observed in practice [3,8]. The PDR link models are shown in Figure 11.

**Figure 12:**
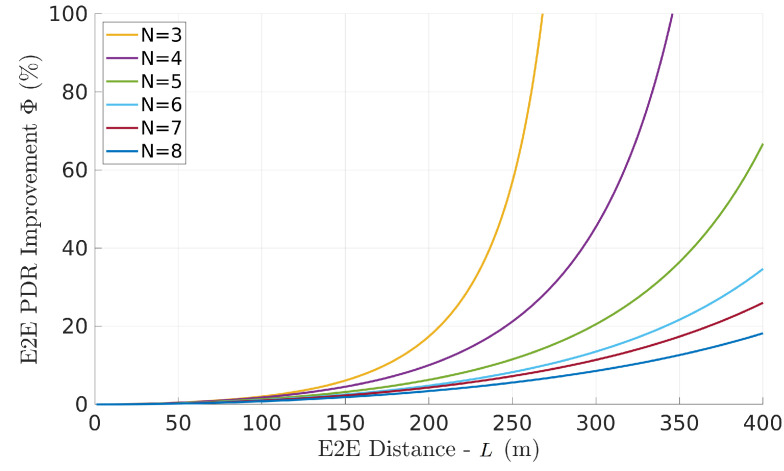
Average network PDR gain Φ (optimal relative to equidistant relay placement) with random link models, as a function of the number of links and network length *L*.

Figure 12 shows the network PDR gain as a function of the number of links (equals the number of relays plus one) and network length in 10,000 scenarios. Each scenario uses a random set of links (link models) generated from our pool. The scenarios are grouped per number of links and averaged. The curves represented in the figure are the average curves for all the scenarios generated with each number of links.

For a given distance, a lower number of links means the average length of each link is longer, thus the overall PDR is generally reduced. In these cases, the optimal placement has a strong impact. Moreover, lower number of links also reveals a faster increase in the optimal placement PDR gain, which comes together with an implicitly faster degradation of the network PDR. This shows that smaller networks (less relays) are more sensitive to the positioning of the relays.

In general terms, as expected, we will observe that the improvement depends on the asymmetry of the links. On the other hand, if all links have the same characteristics, the optimal solution is the equidistant solution and there is no performance improvement.

## 5. Deploying the Optimal Relay Placement

The previous sections gave us, already, good indications of the requirements for operating such a sensing network as well as the benefits that we can expect to achieve. However, to place in practice such a solution we need a deployment strategy that allows operating the relays network to reach the optimal relays positions. In this section we consider two possible approaches, centralized and distributed, which we explain and compare, next.

### 5.1. Centralized Network Control

In this approach, the control architecture relies essentially on the base station, taking advantage of its sink role in the network and potentially higher computing capacity. This approach consists of two phases, a trigger phase, for example, based on tracking the position of the source node, and/or the PDR models of the links. This tracking can also be either centralized or distributed. For consistency with this deployment approach, we assume the source node reports its position and the relays report their PDR measurements from the links they receive from with adequate intervals, e.g., every *x* seconds. Then, the ground station updates its knowledge of the source position and the PDR link models and checks whether these change beyond predefined thresholds and, if so, generates a repositioning event. If this tracking is distributed, the event detection is left to the UAVs that communicate the repositioning events, when they occur, to the base station. A repositioning event initiates the second phase, in which the ground station computes new optimal relay positions and transmits them upstream to the relays as waypoints.

One important aspect is that the total time to reconfigure the network and have the relays taking their optimal positions is dominated by the physics of the UAVs—motion and control. Naturally, this time depends on the distance between the relays starting and target points, which depend on the thresholds used in the trigger phase. With more relaxed thresholds on long networks, this can be several meters apart, taking several seconds to reposition the relays.

Other properties of this approach include the concentration of the computational effort to update the links’ PDR models and compute the optimal relay positions. Naturally, this extra complexity of the ground station is paired with a corresponding simplification of the computational requirements of the UAVs. This solution also implies extra communications downstream that must be always running, even in periods in which the source-sensing stream may not be needed, such as during autonomous source replacement maneuvers. If a distributed event-detection is used, this burden on constant communication is alleviated, but the event communication must be carried out with reliability mechanisms in place.

Finally, this solution falls in the class of event-triggered systems. In the absence of changes in the network, no repositioning event would be generated and no new way-points would be sent to the relay UAVs. Given its simplicity of deployment, we will use this approach as baseline when comparing with the distributed approach that we describe next.

### 5.2. Distributed Network Control

In a distributed context, each UAV places itself optimally with respect to its two neighbors only, thus solely with local knowledge. For this purpose, it tracks the positions of its neighbors and the PDR of the links it is engaged with, and computes its own optimal position only, considering a simple two-links case. Thus, the computational effort required for the UAVs is naturally higher than with the centralized approach, but it is limited, too, and does not depend on the number of relays used. The ground station, conversely, is not involved in the repositioning of the relays, which simplifies its design.

However, the optimization process must be executed iteratively since the positions of all UAVs change, notably the positions of the neighbors that were used to compute the local optimal positions. Therefore, all local optimal positions must be recomputed in each step considering the updated positions of the neighbors. Consequently, we must show that the sequence of local optimal positions converges to the global optimal placements generated by the centralized approach. In other words, given enough time, the final positions generated by both centralized and distributed approaches must be the same. We will address this issue later in this section.

Overall, this solution falls in the class of time-triggered systems in which the positions of the relays are being periodically tracked and adjusted by all relays, in parallel, for adjustment of the PDR models of their links. Thus, changes in relay positions or source position, or variations in links’ PDR, can be compensated readily in an organic fashion, without engaging in global mechanisms. Thus, the line network of relays autonomously tracks its optimal configuration.

#### 5.2.1. Implementing the Distributed Control Method

The implementation of the distributed control method is rather simple, too. Recall that we have *n* links in the network and *n* UAVs of which 2 to *n* are relays. All network nodes regularly share their position with their neighbors, for example, 3D coordinates in an ENU (east–north–up) fixed frame. Consider the relay *i* and its position $r_{i}$. For simplicity, consider that node positions are one-dimensional with origin defined at the source UAV. Its neighbors are the upstream node i−1, with which it shares link i−1 and which is in position $r_{i-1}$, and the downstream node i+1, with which it shares link *i* and which is in position $r_{i+1}$. The PDR models of both links are also known by relay *i*, namely $\left(R_{i-1},\alpha_{i-1}\right)$ and $\left(R_{i},\alpha_{i}\right)$.

Knowing the neighbors’ positions $\left(r_{i-1},r_{i+1}\right)$, relay *i* can apply the optimization method described in the previous section, applied to the simple two links case (Section 4.1.1), to compute its local optimal way-point between its neighbors. This process is repeated with an adequate period *T* as described in the following listing. While all relays from 2 to *n* execute this process, the source UAV and the ground station (sink) simply answer the request for position issued by their neighbor relays. The behaviour just described for relay *i* is summarized in Algorithm 1 (the pseudo-algorithm below).
**Algorithm 1** Pseudo-code for relay *i* in the distributed control method.1:**loop** (every *T*)2:    $\{r_{i-1},r_{i+1}\}=$RequestPosition(nodes {i−1,i+1})  ▹ Get neighbors position3:    $r_{i}^{opt}$ = getOptPosition($\left\{r_{i-1},r_{i+1}\right\},\left\{\left(R_{i-1},\alpha_{i-1}\right),\left(R_{i},\alpha_{i}\right)\right\}$)4:    SetDroneTarget($r_{i}^{opt}$)        ▹ Set waypoint for the local optimal position5:**end loop**6:**procedure**getOptPosition($\left\{r_{a},r_{b}\right\},\left\{\left(R_{a},\alpha_{a}\right),\left(R_{b},\alpha_{b}\right)\right\}$)7:    $\left\{\Psi_{a,b},\theta_{a,b}\right\}\leftarrow f_{Eq13}\left(\left\{\left(R_{a},\alpha_{a}\right),\left(R_{b},\alpha_{b}\right)\right\}\right)$         ▹ cf. Equation (13)8:    $L\leftarrow \left|\right|r_{a}-r_{b}\left|\right|$    ▹ Current length of this network portion9:    $d_{b}\leftarrow \left\{x\in R:\Psi_{a,b}\cdot x^{\theta_{a,b}}+x=L\right\}$10:    $d_{a}\leftarrow L-d_{b}$                 ▹ Compute optimal link lengths11:    $r^{opt}\leftarrow r_{b}+d_{b}$                    ▹ Compute optimal position12:    **return** $r^{opt}$13:**end procedure**

#### 5.2.2. Example

**Figure 13:**
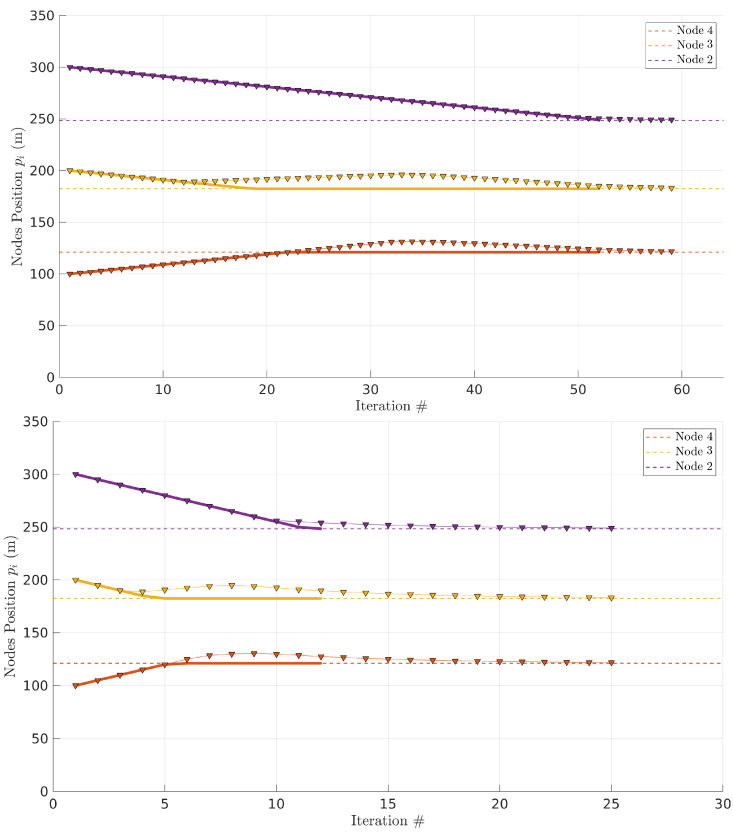
Nodes converging to their optimal position. Triangles represent relay positions every step of the distributed approach. Solid lines show the evolution of a centralized approach. Iterations with *T* = 1 s (**top**) and *T* = 5 s (**bottom**); note the different horizontal scales.

Figure 13 shows the same case with four links (n=4), thus three relays, where the link PDR models were generated randomly (R={154,177,98,108},α={2.53,3.38,2.38,2.59}). The source node is fixed at $r_{1}=L=400$ m from the base station and the initial positions of the three relays are equidistant ($r_{4}=100$ m, $r_{3}=200$ m, and $r_{2}=300$ m, respectively). The horizontal axis shows the number of iterations of the algorithm, repeated with a period of T=1 s (top) and T=5 s (bottom). The vertical axis represents the position of each relay $r_{i}$ along the line between source and base station.

The dashed lines represent the optimal positions for all the relays. The solid lines show the relays’ movement when the global optimal positions are computed at once by the ground station in a centralized approach. In this case, the relay UAVs are assigned way-points corresponding to their optimal positions and move directly to those points at constant cruise speed (1 m/s). Once they reach the optimal positions, they stay there.

The triangles show the relays’ behavior running our distributed approach in which the global optimal locations are not known a priori. We also assume the vehicles move at the same cruise speed of 1 m/s. In this case, the relays share position information with their neighbors every iteration (T=1 s or T=5 s). Note that we are simply executing the algorithms to generate the way-points and consider a simple kinematic model of the UAVs that move when instructed to at constant speed. At 1 m/s speed, this approach is sufficiently accurate for our purposes of comparing the two proposed implementation strategies without resorting to complex simulation engines.

Observing Figure 13, we can see that the centralized approach, despite the immediate computation of the optimal positions, takes 52 s to converge. We define convergence as the time to reposition all relays so that they are at less than 1 meter from their optimal positions. Note that this time is dominated by the physical displacement of the UAVs, not computation time. Curiously, during an initial part of the path, the distributed approach generates sets of local optimal positions to drive the relays that match the UAVs trajectory resulting from the centralized approach. Eventually, there is some bounded divergence for some time, but the distributed approach converges to the global optimal positions. With T=1 s, it takes ∼59 s to converge. This time increases to ∼125 s with T=5 s.

#### 5.2.3. Convergence of Distributed Control

At this point, we have not achieved a formal proof of convergence for the distributed approach, yet. Thus, we tested the convergence hypothesis empirically using 10,000 random scenarios from the same pool of random link PDR models (R,α) used in Section 4.2.2. In each scenario, we compare the time (and track the number of iterations) necessary for the centralized and distributed approaches to converge. We consider two cases with different periods (T∈{1,5} s).

**Figure 14:**
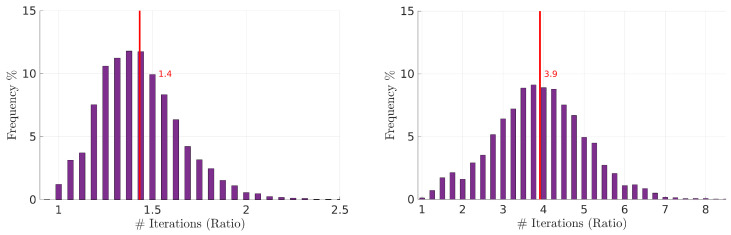
Distribution of the ratio distributed over centralized convergence time for different values of *T*. (**Left**): T=1 s; (**Right**): T=5 s.

Figure 14 shows the histograms of the ratios of the time taken by the distributed approach to converge over the time taken by the centralized approach. We can observe that larger periods take significantly longer to converge. The average ratio grows from ∼1.4 to ∼4 with T=1 s and T=5 s, respectively. Moreover, note that the network is insensitive to changes in positions or link PDR models during each period. Thus, longer periods also generate additional latency in reacting to such changes.

Naturally, sufficiently longer periods will necessarily create instability and prevent the distributed approach from converging. However, we have tried with T=15 s and still achieved convergence, despite taking, on average, ∼8 times the time taken by the centralized approach. On the other hand, setting too-short periods creates additional communication and computing overhead that may turn the network inoperable. Thus, an adequate compromise must be set, considering the desired reactivity to changes, speed of convergence, and communications and computational overhead. Note, however, that the overhead implied by the distributed network control method is low, consisting of one short packet exchange with each neighbor to obtain their positions and one optimal position computation per iteration (every *T*). In such circumstances, we believe that T=1 s is a suitable choice.

### 5.3. On Tracking the Links’ PDR Models

The deployment methods referred before assume the links’ PDR model is known. However, this information is typically unknown a priori. The PDR of each link depends on the area of operation, whether there are obstacles in the area or alien transmitters generating interference, as well as on specific features of the nodes involved, such as their antennas, sensitivity, and noise resilience. Consequently, the links’ PDR must be measured at run time for the estimation of the PDR model parameters (R,α).

This is, in itself, a topic of research and we will not explore it in detail in this work. However, a preliminary strategy has been proposed in [8] and we explain here the basis of its operation. Essentially, the network of UAVs is configured, i.e., defining the number of relays, according to preliminary approximate knowledge of the communication range of the UAVs, possibly taking a conservative approach. Then, the network is deployed at once, with the UAVs being launched in sequence, with the source UAV heading to its initial target and the relay UAVs moving to an equidistant placement between source and sink. This initial travel will take a few seconds, during which the UAV will already exchange a few hundreds of packets (the transmission of the sensing stream is bandwidth intensive, easily reaching 50 to 100 packet/s). All packets are piggybacked with position information and a sequence number. This allows all receivers to build PDR statistics as a function of link distance, for example, every second, allowing to identify the model of each link PDR (since the UAVs are moving, the PDR statistics will consider an average distance during the period in which the packets were collected).

Once the UAVs arrive at their equidistant positions, they will initiate the optimization process to update their positions for maximal PDR. Nevertheless, the PDR measurement and model estimation will continue, always. This process requires a small adaptation of the *RequestPosition* procedure in the pseudocode in Section 5.2.1. In fact, the position from the upstream UAV becomes embedded in the sensing stream; thus, there is no need to ask for it. Moreover, each relay UAV can locally build the PDR model of the upstream link, too. Consequently, the referred procedure just needs to ask the position of the downstream UAV as well as the PDR model of the downstream link to gather the data needed to execute the distributed network control.

## 6. Conclusions

Aerial sensing with multirotor UAVs, in particular, has become common for a myriad of applications, including for remote inspection of difficult-to-access structures in large industrial plants. In this paper, we addressed the case of extending the range of operation of such a UAV, adding aerial relaying support. However, the placement of the relay UAVs is crucial for the end-to-end network performance, notably in what concerns the PDR. We tackled the non-trivial issue of finding the optimal positions that maximize the network PDR in the presence of asymmetrical links. Resorting to simulation, we characterized the average network PDR improvement that can be expected considering a pool of random but realistic link PDR models and how such improvement varies with network length and number of links (or relays). Finally, we addressed the deployment method of the proposed optimization so that the network can be operated in practice. We proposed a fully distributed approach that presents reduced overhead and keeps the relays in optimal positions in an organic manner. With the same pool of random links, we generated 10,000 random networks and executed this distributed approach. When iterated with a period of 1 s, we found that it converges from an equidistant placement to the optimal positions, on average, in 1.4 times the time taken by a centralized method, but without any global coordination. On the other hand, the formal proof of convergence and the online estimation of the links’ PDR model were essentially left for future work.
